# Self-care strategies in response to nurses’ moral injury during COVID-19 pandemic

**DOI:** 10.1177/0969733020961825

**Published:** 2020-10-30

**Authors:** Fahmida Hossain, Ariel Clatty

**Affiliations:** 6613 Duquesne University, USA; 6595 University of Pittsburgh Medical Center, USA

**Keywords:** COVID-19, nurses, self-care, moral injury, moral distress, pandemic

## Abstract

These are strange and unprecedented times in the wake of the COVID-19 pandemic. Most frontline healthcare professionals have never witnessed anything like this before. As a result, staff may experience numerous and continuous traumatic events, which in many instances, will negatively affect their psychological well-being. Particularly, nurses face extraordinary challenges in response to shifting protocols, triage, shortages of resources, and the astonishing numbers of patients who require care in expedited time constraints. As most healthcare workers are passionate nursing professionals, frustration and often a sense of powerlessness occur when they find themselves unable to provide needed care to their patients. The overwhelming number of deaths, patients isolated and dying alone, and the ever-present fear of being infected and then infecting colleagues, family, friends due to the lack of protective gear or known protocols takes its toll on emotional and psychological well-being. For nurses, the experience of this significant (hopefully once-in-a-lifetime) event can inflict on-going moral injury. Nurses affected by this trauma require education, coping tools, and therapy to help avoid or alleviate the adverse effects on their well-being. Institutions must provide these resources to tend to the well-being of their healthcare staff, during and beyond the pandemic. This article aims to investigate moral distress—considering it as a moral injury—and offer tools and recommendations to support healthcare nurses as they respond to this crisis and its aftermath.

## Introduction

COVID-19 has disrupted our medical systems, society, and healthcare practices. The fear that *it* will happen in *our* hospital, fear that *it* will overwhelm within the system, and fear that *it* will return in second and third waves murmurs in the heads of every healthcare worker. This fear has immense impacts on the mental and emotional well-being of medical staff, especially nurses. As a result of the pandemic, the practice of nursing could be significantly changed. This article explains some of the ethical dilemmas nursing faces during this pandemic and the moral distress that arises when caring for critically ill patients with COVID-19. The article explores the overwhelming negative effect serving high numbers of critically ill patients has on nurses. It is high time to more accurately consider this moral distress as moral injury. This article offers recommendations for how nurses can respond within these stressful situations positively by practicing a series of hands-on de-stressing exercises that serve to reduce the moral distress and thus moral injury.

## COVID-19 and its impact on nursing

Nurses are an integral part of the health system. With the uncertainty of COVID-19, nurses face new, daunting, and extreme challenges. In the midst of the pandemic, nurses work long, arduous shifts for extended periods, as a result, many are experiencing severe emotional trauma. Many nurses feel overwhelmed by the massive number of patients needing care, while being immersed in settings full of contagion. As the number of confirmed cases continues to rise, nurses face high-stakes, decision-making challenges that affect both their professional and personal lives.^1,2^

Given the severity of the crisis, a significant shift occurred in terms of ethics. The quick shift from patient-centered ethics to public health ethics presents a major challenge for nurses. Whereas, public health ethics focuses on equity, common good, and the risk and benefit to society as a whole, patient-centered ethics focuses on duty to care for a particular patient. The focus of public health ethics is the greater good for society while patient-centered ethics focuses on the individual patient’s need.^3^ Within a few short weeks, the nursing profession was directed to apply public health as the guiding dictate when caring for patients. This transition is tough, cumbersome, and rubs against many basics of nurse training. Particularly in the United States, nurses train to provide patient-centered care or an autonomy-driven approach. During the pandemic and given the scarcity of resources, nurses often have no other choice but to be paternalistic in their practices. Nurses become directive in allocating resources and their time. Even though a patient may want more comforting care, nurse must leave this patient unattended to serve another patient who is more vulnerable. Situationally, and in short order, nurses have become directive decision-makers, which runs counter to their ethical directives, and the values instilled by modern patient-centered medicine.^4^

Nurses who routinely face difficult situations, now confront situations that most have never experienced before. Nurses are called on to provide end-of-life care to COVID-19 patients without the patient’s family and loved ones present. Closure for family and friends is delayed or does not occur. Patients are relegated to FaceTime and other video means as ways to communicate, and often to say goodbye, if at all. Nurses are placed in the position to tell families they cannot visit. Nurse are called on to enforce new protocols and policies. More so, nurses find themselves anxious of carrying the weight of being “family” to patients, many who will not survive the virus.^5^ These changes, and particularly taking on the role of surrogate “family” give rise to distress that can become injurious to the caregiver. In the maelstrom, it becomes more difficult for nurses to separate what needs to be done and what ought to be done. Nurses thus experience moral injury, which is a long-lasting psychological and emotional effect that arises from actions taken that run in opposition to one’s personal moral values or beliefs. Moral injury occurs when people fail to uphold their moral values or beliefs. Nurses are morally and ethically obligated to provide patients with the best treatment.^6^ However, due to the increased number of patients, nurses are unable to provide the best care, or the care the patient needs. This takes a toll on nurses. A study conducted in 2016, during “normal” times in intensive care unit (ICU) care, the most commonly reported causes of moral distress were concerns about the care provided by other health care workers, the amount of care provided, poor communications, inconsistent care plans, and issues around end-of-life decision making.^7^ These issues still exist within this crisis situation; however, the added stress of triage and implementing care faster and in fragments exacerbates any previous problems within ICU care.

## Ethical dilemmas in nursing

In this altered landscape, nurses confront choices that are often morally and ethically challenging, such as:

### Fairness and justice

During the COVID-19 pandemic, even the wealthiest countries have relied on triaging as patient demand overwhelmed available resources. However, the practice of triage and prioritization gives rise to ethical questions. During this chaos, nurses have to decide whom to provide care and resources, and for how long to tend to this patient. These are perplexing ethical and moral dilemmas.^7^ In parts of the world, the number of patients hospitalized due to COVID-19 patients have depleted the available supply of ventilators. Despite the lack of resources, every nurse continues to have an obligation and duty to treat the patient at hand. Nurses find themselves forced to make moment-to-moment ethical decisions on who should receive treatment. Who should be seen first, who should be seen later? How much time to allocate at the bedside? How to address the grief? All these choices confront core ethical questions which revolve around preserving the life and dignity of the individual patient. This situation is extremely stressful. Such choice-making often falls outside the realm of nurses’ training and expertise.^8^

### Duty to care

Nurses are trained to care for patients with empathy and expertise. However, due to a lack of resources, patient-centered care is not always possible. Nurses are asked to make tough ethical decisions, which run contrary to their training and the core human concern for the other’s well-being. With the debilitating nature of COVID-19 and its deadly intensity, nurses are forced to follow the guides of public health ethics, some of which are significantly different than those of patient-centered-ethics of care. Public health ethics holds the welfare of the community as a greater good than the well-being of an individual patient. Whereas in care ethics, a nurse’s obligation is to care for the patient at hand and serve through the “actual giving of care” while respecting the dignity and worth of each patient. Moral distress occurs when nurses cannot follow their creed; COVID-19 places nurses in situations where they are forced to choose one patient’s well-being over that of another. As a result, nurse are unable to provide due care to all equally.^9^

Being forced to make clinical decisions in the face of limited resources is a heavy burden for nurses to carry. It is emotionally challenging to force a nurse to pick between what is morally right and what is viable or affordable in a given situation. The tension arises when deciding between individual needs versus community needs.^10^ Also, a nurse is obligated to provide compassionate care to each patient. However, during this pandemic, nurses are simply unable to provide this level of care due to time, resources, and facility constraints. As a result, many nurses are taken by a sense of helplessness, question their abilities, and are forlorn and frustrated at the bedside of a patient who is cut off from family and friends, dying alone.^11^ “That’s a tough thing to watch every single day, to watch somebody die without their family there,” said Jennifer Mueller, RN. Her statement clearly and succinctly reveals the trauma she and many nurse are going through.^12^

The inability to provide care to all presents near-insurmountable ethical dilemmas. Nurses struggle mightily to adapt to this “new normal.” Many have difficulty responding to the excessive traumatic stress they face and, as a result, may develop post-traumatic stress as a byproduct of making untenable ethical decisions. Nurses need support, coaching, and reassurance that “the less than the adequate care” given in the name of social welfare is not unethical or done to harm individual patients intentionally.^13^

### Personal safety versus professional integrity

Nurses, like all people, have families and loved ones in their lives. Nurses find themselves with competing obligations to work, family, and loved ones. This dilemma is a conflict between professional obligations and personal responsibilities. How can nurses balance or make peace with this predicament? On the one hand, there are obligations to virus sickened patients staring at them for help and assurance. And on the other, the embodied obligations to those they love and depend on in so many common ways.

Beyond these ethical challenges, the scarcity of personal protective equipment (PPE) adds further confusion into the decision-making landscape nurses are facing. The United States, as do many other western countries, struggles to provide adequate protection to frontline workers. This gives rise to another ethical dilemma nurses confront: nurses face the obligation to care for patients, but they also have the right and responsibility to care and protect themselves and their families.^14^

### Re-narrated job description

Most nurses in the United States and other wealthy countries have little experience practicing medicine in a compromised, overwhelming situation like the one COVID-19 has created. During their education and clinical training, nurses generally have ample resources and support at hand. Nurses are not trained for situations like the one in which they are now immersed. Many nurse practitioners find themselves overwhelmed, uneasy, confused, scared, angry, yet committed to their nursing roles—often while compromising their health and that of those they love. Furthermore, nurses (and all staff) find themselves as innovators, problem-solvers, and sometimes mechanics, finding creative ways to stretch or re-allocate scarce resources, or re-purposing one thing to do another.

As example, nurses have designed and constructed low-cost, easily made and mass producible protective shield to safeguard front liners. Nurses determined to provide care faced safety challenges. In response, they began designing and making PPE for the front liners. Applying insight gained from handling Ebola patients and using their knowledge, expertise, and creativity they constructed a lightweight protective shield. Eighty-one thousands of these shields have been distributed. Another group of creative nurses implemented virtual rounds for the families of patients who could not visit in person due to COVID-19 restrictions. They called their virtual visits “Real Talk Real Time” and brought the families and patients together. Another nurse invented a “code card” to help the care team to provide important messages in a quick and effective manner in a patient room, saving others from entering and risking infection. The idea for the code card came to her after she experienced her first code blue incident at an ICU. These are only a few of the numerous examples of nurses as active innovators, problem-solvers, and leaders. In the face of COVID-19-related challenges, nurses have naturally taken on the role of just-in-time innovators.^15^ Some nurses have also found ways to use digital technologies to ensure social distancing in hospitals.^16^

The crisis came with no playbook that accounts for the intensity of the illness or the lack of resources. Thus, nurses are creating new or adapting current practices as means to tend to those whom they find in need of care. Nurse are learning, creating, applying new skills and processes on the fly within the middle of the crisis. The innovations, give nurses sense of accomplishment. However, the added roles nurses take on create ongoing ethical challenges in terms of their professional integrity and gives rise to moral distress.^17^

## Moral distress and moral injury

Many nurses and healthcare professionals are likely to experience post-traumatic stress as a consequence of serving during the COVID-19 crisis. The ethical dilemmas mentioned above, alone or in combination, can lead to severe moral distress. Moral distress occurs when a person acts in a way that goes against an established ethical and moral response to a situation. Institutional and structural limitations have placed nurses in positions where they must make a series of decisions, shift after shift, minute by minute, which run counter to their training, responsibilities, and, often, personal beliefs. Repeatedly, these decisions are made knowing they do not best serve a particular patient.^9^ As a consequence of COVID-19, a variety of mental health problems are surfacing, leading many healthcare professionals to seek counseling and support services. Tragically, there has also been a spike in suicides among healthcare professionals in the midst of this pandemic.^2,7^

Nurses are critical to the administration of excellent care. They are even more focal during this crisis because they play expanded and multiple roles simultaneously: performing diverse roles, conducting screening processes, attending to the critically ill, deciding triage protocols, contacting and updating families, and informing the family of the death of a loved one. In many ways, moral distress in this situation might be better seen as moral injury. These moral injuries may be long-lasting due to the intensity of the crisis. Post-traumatic responses are highly likely as a result. During this crisis, many nurses struggle to share with others the effect of seeing someone die, knowing the reality of the situation did not permit them to provide the care that was needed or necessary. Here is where the seeds of moral injury are sown.^18^

Moral injury is the term that is used in the military. Moral injury is a long-lasting emotional, psychological, social, and spiritual effect from actions taken that run contrary to one’s moral values.^18^ The stress the nurses experience will not only create moral distress but will have a lasting impact. This is why the term moral injury is best used in the context of COVID-19. However, some scholars argue that the term moral injury is inappropriate for medical professionals. The argument is the scenarios in war and the scenarios in medical practice are significantly different.^19^ However, the gravity and the complexity of COVID-19 have erased many of the distinct differences between military and medical workers’ experiences. Questions that must be addressed, even in chaotic hospital settings, frequently depicted through battlefield and war analogies. The volume of deaths and the number of critical patients give rise to battlefield and war metaphors to describe the clinical scene. Regardless of terminology, the results seem to be the same or eerily similar. Given the duty nurses take on during this crisis, there are clear and abundant signs of psychological distress that must be recognized and addressed.

Nurses may find difficulties in translating their moral decisions into moral action due to the lack of sufficient healthcare ethical training and education. To address these issues, ethical training is necessary to educate student nurses and new nurses on ethical concepts and methodologies to recognize and respond knowingly and appropriately when encountering ethically challenging situations.^20^

## Self-care strategies

Healthcare professionals are often termed HEROs (High Expectation and Risk Occupation).^11^ The profession broadly, and the organizations that employ them, must be well aware of the needs of HEROs. In the near and long-term, nurses will require support and help as they make sense of the crisis and reshape their lives, new normal or not. HEROs must know that self-care is not selfish, but smart. The effects of distress are cumulative. It must be understood that if a provider is not well, it becomes challenging to heal others without doing further harm to oneself.

When moral distress is not addressed it can lead to burnout, feelings of frustration, and chronic exhaustion. Unattended stressors can lead to secondary traumatic effects which are identified as negative feelings, vicariously acquired due to indirect exposure of trauma-related events.^21^ If nurses do not have proper education, training and tools to mitigate this trauma and this pandemic continues long term, nurses will be ill prepared to respond to the psychological effects that could lead to burnout and turnover, or worse. Nurses who experience burnout may experience impaired emotional and physical health and a diminished sense of well-being. This heavily effects the severity of moral injury. High levels of burnout signify that nurses possess insufficient resources to respond to the demands of their jobs, which often leads to impaired job performance.^22^

### Moral resilience

One way to self-care is by building moral resilience. Self-efficacy and self-control help nurses respond positively to distress they encounter as nurses.^23^ Moral resilience is the courage and confidence to confront distressful and uncertain situations by following and trusting values and beliefs. Being morally resilient allows one to maintain perspective, keep a situation in context, and understand that some conditions are out of one’s control. From that position, the nurses can precede without seeing themselves as deficient, not doing enough, weak, or neglecting others.^24^ Moral resilience can be built and developed, for instance, by practicing mindfulness. Being mindful helps nurses reduce the cases of distress, anxiety, fear, and helplessness that occur through the trauma of COVID-19 clinical settings. Nurses can also strengthen their parasympathetic nervous systems to combat stress through breathing exercises and mindfulness.

Mindful breathing is also helpful before entering into a patient’s room as a means to calm oneself from the previous encounter.^10^ There are self-care and breathing Apps, such as Calm or Headspace, to help a nurse stay attuned to develop moral resilience.^25^ Building on moral conscientiousness, moral resilience includes the ability to make important ethical distinctions, remain open-minded and curious, and to resist the presumption that there is only one way to consider one’s moral obligations or to preserve integrity in any particular situation.^24^ Moral resilience can help nurses find meaning and respond to ethical issues in a constructive, positive, and healthy way.

### Self-stewardship

Self-stewardship is the skill of tending to and nurturing one’s well-being. Without self-stewardship it becomes challenging to stay healthy and to serve others well. Self-stewardship—allowing oneself to be seen—helps nurses to contextualize the ethical dilemmas they face between patient-centered care and public health ethics. This helps an individual understand and appreciate that she does nothing “wrong” by providing public-health guided care.^26^ Psychological interventions and support provide structured and profession forums in which care professionals can talk through and contextualize the ethical and personal challenges and uncertainties they face. For instance, providing care while wearing a mask, face shield, and other PPE is extremely frustrating. However, having a defined space or forum to express such everyday frustrations to colleagues or psychologist can create safer, more relaxed, and positive environments.^10^

## Structural support

Apart from self-care, each individual at every level of the organization must understand the negative effects that the COVID-19 crisis has upon nurses. Discounting or ignoring the mental health of nurses will have some adverse short- and-long-term consequences for the healthcare delivery system. Coworkers and institutional leaders must recognize the prevalence and magnitude of moral distress and stand together to view nurses as individuals in need. It important not to look at nursing in the abstract or as statistics.^27^ From the organizational stand point, management and coworkers should be highly visible and encouraging of nurses, ensuring them that that “it is ok” to be emotionally vulnerable. Nurses are typically uncomfortable sharing their feelings with others, but now is the time to make it comfortable for them to open up. Otherwise, the physiological wounds and scars will be more severe.

During the crisis, it is important to acknowledge the successes achieved by nurses, no matter how small. The goal is for a nurse to stop the “yes, but” response to praise given; nurses must learn to accept and acknowledge praise. It is imperative that all providers help nurses acknowledge what went well. Such acknowledgments help bring light into the darkness that seem to cloak the chaos of the crisis. Beyond acknowledgment, hope and a sense of accomplishment must be cultivated by the organization. Instilling hope—and a belief that the crisis will improve, that the future will be better—into the fabric the organizational culture can bring a remarkable change in mood, safety, mental health, and performance.^10^ Organizations must fully acknowledge the stress and burdens faced by providers. Offering hope is a means to keep the community together, and to keep nurses focused and intent on collectively overcoming the challenges they face. Perfectionism must be cast aside. It is unattainable and only leads to unrealistic expectations. The COVID-19 crisis is a challenge which requires nurses to accept ambiguity and uncertainty while honoring themselves by embracing their humanness.

Nurses also need support and education as they try to work to regain “normalcy.” Cognitive processing therapy is a form of cognitive behavioral treatment to help victims of trauma. There are four main steps which include education, information, developing skills, and changing beliefs.^28^ Through this, trauma therapists can help the nurses to identify possible symptoms of posttraumatic stress disorder (PTSD) and lead them to understand how receiving treatment can help. The therapist, in turn, can help staff recognize how their thoughts and feelings are related directly to the stress and anxiety they are experiencing. Nurses should be given training and skill building opportunities provided by the institution that offer coping mechanisms. This will help nurses question or challenge their beliefs and routines that do not serve them well during the distressing and morally complicated situations that they face.^29^

Another type of therapy that has shown positive results is emotional freedom techniques (EFT). This type of therapy combines cognitive behavioral therapy and exposure therapy. This type of therapy may also involve a type of acupuncture:

During a typical treatment session, clients recall a traumatic event and pair it with a statement of self-acceptance, such as “Even though I experienced [name of the event] I deeply and completely accept myself.” They stimulate 7 acupuncture points with their fingertips (acupressure) by tapping on them or rubbing them while repeating the name of the event.^29^

By engaging in active monitoring and trauma focused cognitive behavioral therapy, medical staff can also follow and apply the United Kingdom’s National Institution for Health and Care Excellence (NICE) guidelines for clinical psychological disorders and treatment standards. The implementation can be achieved through a controlled peer support program that has been adopted by many institutions like the military or emergency services that are involved in trauma situations.

The goal of these therapies is to ensure that the mental health of nurses is prioritized and attended to. Regardless of approach, the aim is to address and alleviate emotional, psychological, and the embodied distress nurses encounter. Whether the therapies are structured interventions, or quiet spaces away from the fray, positive benefits occur for the nurses when they recognize or acknowledge the challenges they are experiencing.^10^ These approached provide pathways for nurses to address and respond to their emotions in proactive ways and in supportive environments in the midst of the high-pressure crisis. Addressing this trauma contemporaneously makes tending the effects of mental and physical trauma normal and it begins to be seen as a best practice. Thus, nurses who are aware of their emotions, feelings, and distress are more likely to open up and supportively address the diseases confronting them.^30^

There are many ways that healthcare organizations can enhance and improve their organizational culture and moral resilience when dealing with traumatic events. It is important for the organization to focus on improving their social support and leadership in order to sustain everyday tasks in the new normal. This is especially important post trauma. It is important for leadership and management staff to understand when their floor staff is going through post trauma therapy that their work duties may slightly change. When someone is treated for PTSD, she will likely benefit by avoiding high-stress situations, and may need to alter the working hours depending on concentration, irritability, and avoidance.^31^ The goal is to create a safe work environment and to build a culture of resilience with avenues of support.

Healthcare institutions are obligated to meet the needs of patients, but also the needs of staff. Healthcare staff will continue to put the needs of their patients before their own, and may not recognize that they too need to be cared for. It is the duty of the institution to provide the tools that serve to keep staff safe and protected, including their mental and emotional components of their health. Organizations should offer trauma therapy in order to attend to their nurses’ emotional and psychological needs during this pandemic. These are just a few types of therapy opportunities that institutions can engage in.

## Conclusion

These are strange and unprecedented times we currently face. While the majority of the people will “shelter in place” during the pandemic, we cannot let the nurses “shatter to pieces.” Without proper attention, hospitals will face mental health emergencies among nurses when the COVID-19 pandemic has subsided. The number of suicides will rise and PTSD will be prevalent and common among nurses.^1,2^ The unresolved moral distress has been correlated with burnout and long-term consequences such as emotional exhaustion, depersonalization, feelings of disengagement, numbness, and diminished moral sensitivity.^32^ These, in turn, are forms of moral injury. It is high time to adopt self-care strategies to combat the moral injuries of our nurses and support them through this difficult time. Approaches to developing moral resilience, self-stewardship include “rewiring the brain,” ethics education, hospital education, and organizational support that should be offered to nurses experiencing crisis in COVID-19. Without this type of support, there will be increasingly high rates of moral distress, moral injury, and burnout.
